# Domains of quality of life in Alzheimer’s disease vary according to caregiver kinship

**DOI:** 10.47626/2237-6089-2019-0036

**Published:** 2021-02-26

**Authors:** Marcela M. L. Nogueira, Jose Pedro Simões, Marcia C. N. Dourado

**Affiliations:** 1 Centro de Doenças de Alzheimer e outras Desordens Mentais na VelhiceInstituto de PsiquiatriaUniversidade Federal do Rio de JaneiroRio de JaneiroRJBrazil Centro de Doenças de Alzheimer e outras Desordens Mentais na Velhice, Instituto de Psiquiatria, Universidade Federal do Rio de Janeiro, Rio de Janeiro, RJ, Brazil.; 2 Departamento de Sociologia e Ciência PolíticaCentro de Filosofia e Ciências HumanasUniversidade Federal de Santa CatarinaFlorianópolisSCBrazil Departamento de Sociologia e Ciência Política, Centro de Filosofia e Ciências Humanas, Universidade Federal de Santa Catarina, Florianópolis, SC, Brazil.

**Keywords:** Quality of life, kindship, caregivers, spouse-caregiver, Alzheimer’s disease

## Abstract

**Introduction:**

Compared to other types of caregiver, spouse-caregivers tend to be closer to people with Alzheimer’s disease (PwAD) because of their different position in the relationship. We designed this study to compare the differences in caregivers’ quality of life (QoL) and domains of QoL according to the kinship relationship between the members of caregiving dyads.

**Methods:**

We assessed QoL of 98 PwAD and their family caregivers (spouse-caregivers, n = 49; adult children, n = 43; and others, n = 6). The PwAD and their caregivers completed questionnaires about their QoL, awareness of disease, cognition, severity of dementia, depression, and burden of caring.

**Results:**

The comparison between caregiver types showed that spouse-caregivers were older, with higher levels of burden and lower scores for cognition. Caregivers’ total QoL scores were not significantly different according to type of kinship. However, there were significant differences in the domains physical health (p = 0.04, Cohen’s d [d] = -0.42), marriage (p = 0.01, d = 1.31), and friends (p = 0.04, d = -0.41), and life as a whole showed a trend to difference (p = 0.08, d = -0.33). When QoL domains were analyzed within dyads, there were significant differences between members of spouse dyads in the domains energy (p = 0.01, d = -0.49), ability to do things for fun (p = 0.01, d = -0.48), and memory (p = 0.000, d = -1.07). For non-spouse dyads, there were significant differences between caregivers and PwAD for the QoL domains memory (p = 0.004, d = -0.63), marriage (p = 0.001, d = -0.72), friends (p = 0.001, d = -0.65), and ability to do chores (p = 0.000, d = -0.76).

**Conclusions:**

Differences were only detected between spouse/non-spouse-caregivers when QoL was analyzed by domains. We speculate that spouse and non-spouse caregivers have distinct assessments and perceptions of what is important to their QoL.

## Introduction

Depression, anxiety and stress are common among the caregivers of people with Alzheimer’s disease (AD). Several studies have investigated how these factors affect caregivers’ quality of life (QoL) and sense of burden, but these studies commonly include different types of caregivers, such as spouse, adult children, and other relatives who take care of people with Alzheimer’s disease (PwAD).^1,2^ Questions therefore remain in relation to whether there are differences in caregivers’ perspectives on QoL according to the degree of kinship between caregiver and care receiver.

A meta-analysis comparing different types of caregivers showed that, because of their closer relationship with the PwAD, adult children caregivers report more depression and psychological distress related to caregiving when compared to children-in-law caregivers.^3^ Moreover, caregivers who lived in the same home with the PwAD had lower QoL compared with those who did not.^4^

In AD, when spouse-caregivers are compared to those with other types of kinship, they often feel more responsible for the caring activities and, consequently, present higher risk for feelings of burden and depression.^3^ Spouse-caregivers perceived their role as being more stressful than non-spouse-caregivers,^5^ and this may influence their evaluation of QoL. Although each member of the couple experiences different changes in the relationship, these data may be explained by hours of caring, change in intimacy, and generally older age.^3,6^ Furthermore, the relationship may also be influenced by changes in sexual activity. Commonly, PwAD may not be able to remember what to do during sexual intercourse and/or exhibit inappropriate sexual behaviors, causing the spouse-caregiver to experience feelings of rejection.^7^ Thus, the level of intimacy of the spouse-caregiver/PwAD couple may influence their QoL differently. If a spouse-caregiver perceives the relationship as less distressing, the quality of care provided will be higher, resulting in higher QoL for the couple.^6^ As a result, the quality of the marital relationship may be associated with QoL.^8^ A perceived change in relationship was therefore found to be an important determinant of caregiver QoL.^4^

Quality of life is a complex and multidimensional concept that comprises subjective and objective indicators, such as interpersonal relationships, family relationship, levels of chronic illness, housing, and changes to feelings.^6,9^ In dementia, QoL includes four general domains: cognitive functioning, activities of daily living, social interaction, and psychological well-being.^9^ According to Novelli et al.,^10^ the importance of evaluating QoL lies in determining which interventions benefit PwAD and their caregivers over the course of disease progression. On the other hand, what is important to evaluate regarding QoL may not be the same for different kinds of caregivers and PwAD, even when all are asked the same questions.^11^ Therefore, to better understand the influence that the type of relationship between PwAD and their caregivers has on QoL and its domains, we decided to compare the differences in caregivers’ perspectives according to the degree of kinship between dyads. Thus, this study aims: 1) to compare the influence of different kinships on caregivers’ self-report QoL ratings; and 2) to compare the differences between PwAD self-report QoL domains and caregiver self-report QoL domains according to their degree of kinship. We hypothesized that QoL is lower in spouse-caregiver dyads than in non-spouse-caregiver dyads and that overall PwAD self-report QoL scores are higher than caregivers self-report scores.

## Methods

### Participants

The sample comprised PwAD (n = 98; 43 males) diagnosed according to the Diagnostic and Statistical Manual of Mental Disorders, 4th edition (DSM-IV),^12^ and their family caregivers (spouse-caregivers, n = 49; adult children, n = 43; and others, n = 6). The PwAD had attended an outpatient clinic in the city of Rio de Janeiro, Brazil, for routine follow-up appointments and were consecutively selected after psychiatrist referrals, from January 2016 to May 2018. Clinical diagnosis of AD was based on a clinical interview with the PwAD and caregiver, cognitive screening tests, laboratory tests, and imaging studies. Only individuals with Mini-Mental State Examination (MMSE)^13^ scores of 13-26 and classified as Clinical Dementia Rating (CDR)^14^ 1 or 2 according to total score were included in the study.

In order to ensure that findings would be applicable to AD rather than other neurological problems, PwAD with history of head trauma, aphasia, alcoholism, psychiatric disorders, or epilepsy were excluded from the study. All PwAD were able to read and write simple sentences, interact with the interviewer, and understand and follow instructions.

Caregivers were classified into two categories: spouse-caregivers and non-spouse caregivers who were nonetheless the person primarily responsible for the’s care. Only informal caregivers were included in the sample (i.e., family members or volunteers). Non-spouse caregivers who did not live with their charges should have been visiting them at least once to twice a week, in order to be able to comment on actual daily living behaviors and challenges. We excluded caregivers with a reported history of psychiatric or cognitive disorders. Only caregivers with MMSE scores of 28-30 were included in the study. All caregivers were able to provide detailed information about their charges. All caregivers had been previously informed of the diagnosis by the psychiatrist. Each member of the PwAD-caregiver pair was interviewed separately from the other member of the pair.

The PwAD completed assessments covering QoL, cognition, and awareness of their disease. The caregiver provided information about the PwAD (including demographic data, depressive symptoms, and dementia severity), their own QoL, and the perceived level of the burden of care.

This study was approved by the ethics committee at the Instituto de Psiquiatria – Universidade Federal do Rio de Janeiro (UFRJ), and all of the PwAD and caregivers were given a full description of the study and signed informed consent forms prior to the first interview.

### Measures

All instruments used were validated versions adapted for Brazil. All instruments and procedures were applied in a standardized manner and in the same sequence by trained researchers.

#### PwAD measurements

Quality of life. The primary outcome was QoL measured with the Quality of Life in Alzheimer’s Disease Scale (QoL-AD). We used the self-report version of the scale (QoL PwAD). The scale evaluates 13 specific domains (physical, health, energy, mood, living situation, memory, family, marriage, friends, chores, fun, money, self, and life as whole) which are rated as: 1) poor, 2) fair, 3) good, or 4) excellent. The score ranges from 13 to 52, with higher scores indicating higher QoL.^10^

Cognition. The MMSE is an instrument that comprises tests of orientation, memory, attention, ability to name, and ability to follow verbal and written commands. The total score ranges from 0 to 30. Lower scores indicate impaired cognition.^13^

Dementia severity. The Clinical Dementia Rating (CDR) scale was used to measure AD severity, with stages ranging from 0 (no dementia) to 3 (severe dementia) according to cognitive, behavioral, and activities of daily living impairment). We used the full protocol.^14^

Awareness of disease. The Assessment Scale of Psychosocial Impact of the Diagnosis of Dementia (ASPIDD) is a 30-question scale based on the reports of PwAD and their caregivers. This scale was designed to evaluate awareness of disease by scoring discrepant responses across domains that include cognitive functioning and health condition, activities of daily living, emotional state, social functioning, and relationships. The caregiver answers the same questions as the PwAD. The score is based on the degree of discrepancy between the PwAD’s and caregivers’ responses, with one point being scored for each discrepant response. Awareness ratings range from preserved (0-4), mildly impaired (5-11), moderately impaired (12-17), to absent (over 18).^15^

Depressive symptoms. The Cornell Scale for Depression in Dementia (CSDD) was used to measure PwAD circadian functions, and physical, mood, and behavioral symptoms. The maximum score is 38 and a cut-off score of 13 indicates presence of depression.^16^

#### Caregivers’ measurements

Quality of life. The same scale used to assess PwAD QoL was also administered to the caregivers (QoL C).^10^

Cognition. This is the same scale (MMSE) used to assess PwAD cognition, but only caregivers who scored between 28 and 30 were included in the study sample.^13^

Burden. The Zarit Burden Interview was used to measure caregivers’ perceived level of burden. Its 22 closed questions assess the impact of the illness on the caregiver’s life. The score ranges from 0 (no burden) to 88 (high burden).^17^

## Statistical methods

All data and statistical analyses were performed with SPSS version 21.0. Clinical and sociodemographic characteristics of PwAD and caregivers were analyzed with descriptive statistics. Parametric statistics were calculated using mean and standard deviations (SD). Non-parametric variables are expressed as the percentage of the highest option.

The Levene test was used to test for homoscedasticity of variances. The Chi-square and Fischer’s exact tests were used to assess differences in gender and educational level. The Mann-Whitney U test and Kruskal Wallis test were used to verify heterogeneity of variances. The paired Student’s *t* test was used to assess differences between dyads’ QoL ratings (physical health, energy, mood, living situation, memory, family, marriage, friends, chores, fun, money, self, and life as a whole). We corrected for multiple comparisons (Bonferroni correction). Only variables with p < 0.005 (p < 0.05/9, corrected for multiple comparisons) were included.

We used Cohen’s d (d) to measure effect size when comparison of two means in the domains of the QoL-AD scale revealed significant differences. The values indicated weak (< 0.5), moderate (0.5-0.8), or high (> 0.8) effects.

All significance tests were performed at a two-tailed α level of ≤ 0.05.

## Results

### Description of the sample

The mean age of PwAD cared for by spouses was 75.9 years (SD = 8.1) and mean age of PwAD cared for by non-spouses was 77.9 years (SD = 5.7). The majority of the spouse PwAD were male (n = 35) and the majority of non-spouse-PwAD were female (n = 40).

The mean ages of caregivers were 69.1 years (SD = 10.1) for spouse-caregivers and 50.8 years (SD = 9.5) for non-spouse caregivers. The most prevalent type of non-spouse caregiver kinship was adult child (88%, n = 43). Sociodemographic data for dyads are shown in Table 1.

**Table 1:** Sociodemographic and clinical characteristics of PwAD and their caregivers

	Spouse-PwAD (n = 49)	Non-spouse-PwAD (n = 49)	p	Spouse-caregiver (n = 49)	Non-spouse caregiver (n = 49)	p
Gender, n (%)						
Male	35 (71)	9 (18)	< 0.001^‡^	14 (29)	7 (14)	0.11
Female	14 (29)	40(82)		35 (71)	42 (86)	
Age (years)	75.9 (8.1)	77.9 (5.7)	0.33	69.1 (10.1)	50.8 (9.5)	< 0.001^‡^
Age at onset	71.3 (9.4)	72.5 (5.8)	0.28	-	-	-
Educational level (years)	8.6 (3.9)	6.5 (3.3)	0.003^†^	10.3 (3.5)	12.6 (2.4)	< 0.001^‡^
CDR, n (%)						
Mild	33 (67)	33 (67)	0.58	-	-	-
Moderate	16 (33)	16 (33)		-	-	-
MMSE	20.4 (4.6)	18.8 (3.7)	0.06	28.1 (1.2)	29.0 (0.8)	0.01*
ASPIDD	9.3 (6.0)	8.5 (3.4)	0.40	-	-	-
ZBI	-	-	-	33.3 (15.0)	24.7 (13.5)	0.02*
CSDD	9.0 (5.8)	7.4 (5.2)	0.26	-	-	-

Data presented as mean (standard deviation), unless otherwise specified.

ASPIDD = Assessment Scale of Psychosocial Impact of the Diagnosis of Dementia; CDR = Clinical Dementia Ratings; CSDD = Cornell Scale for Depression in Dementia; MMSE = Mini-Mental State Examination; PwAD = people with Alzheimer’s disease; QoL = quality of life; ZBI = Zarit Burden Interview.

* p < 0.05; ^†^ p < 0.01; ^‡^ p < 0.001.

### Clinical data

PwAD. The majority of PwAD showed mild disease severity, with 67% (n = 33; 19 male) of spouse-PwAD and 67% (n = 33; 4 male) non-spouse-PwAD reporting mild severity.

Regarding awareness of disease, 24% (n = 12) of spouse-PwAD dyads reported preserved awareness, as compared to 16.5% (n = 8) for non-spouse PwAD; mildly impaired awareness was observed for 43% (n = 21) of spouse-PwAD and 63.5% (n = 31) of non-spouse-PwAD. Moderately impaired awareness was observed for 23% (n = 11) of spouse dyads and 20% (n = 10) of non-spouse PwAD. Interestingly, 10% (n = 5) of spouse-PwAD were unaware of their disease. Total score for awareness was 9.3 (SD = 6.0) for spouse-PwAD and 8.5 (SD = 3.4) for non-spouse-PwAD (p = 0.40; d = 0.17).

Caregivers. There were significant differences between spouse and non-spouse caregivers in cognition (p = 0.01; d = -0.90) and level of burden (p = 0.02, d = 0.60). Non-spouse caregivers scored higher for cognition and scored lower for burden.

Clinical characteristics of PwAD and caregivers are shown in Table 1.

### Quality of life

Differences between self-report caregivers’ QoL domains according to degree of kinship. Caregivers’ total QoL scores were 36.4 (SD = 5.1 – range 31.3-41.5) for spouse-caregivers and 36.7 (SD = 6.6 – range 30.1-43.3) for non-spouse-caregivers. The non-spouse-caregivers scored slightly higher for QoL than spouse-caregivers, but the difference was not statistically significant (p = 0.65, d = -0.05). However, there were significant differences in QoL in the domain of marriage (p = 0.01, d = 1.31), as expected, with a large effect size. Additionally, physical health (p = 0.04, d = -0.42) and friends (p = 0.04, d = -0.41) were significantly different and there was a trend for different life as a whole scores (p = 0.08, d = -0.33), all with small effect sizes.

Differences between self-report PwAD and self-report caregivers’ ratings of QoL. There was a significant difference between spouse-PwAD and spouse-caregivers self-report QoL (p = 0.01, d = -0.40), with a small effect size. Spouse-caregivers scored higher for QoL than spouse-PwAD.

When the domains of QoL were analyzed, there was a significant difference in energy (p = 0.01, d = -0.49) and ability to do things for fun (p = 0.01, d = -0.48) with a small effect size. However, differences in the memory domain (p = 0.000, d = -1.07) revealed a large effect size.

Non-spouse caregivers reported higher self-report QoL than spouse PwAD, with a significant difference between groups (p = 0.006, d = -0.54) and moderate effect size.

There were significant differences in the QoL domains memory (p = 0.004, d = -0.63), marriage (p = 0.001, d = -0.72), friends (p = 0.001, d = -0.65), and ability to do chores (p = 0.000, d = -0.76), all with medium effect size.

The differences in QoL domains and total score are shown in Tables 2 and 3.

**Table 2:** Differences in self-report QoL domains for spouses and non-spouses

QoL	Spouse-PwAD	Non-spouse-PwAD	p	d	Spouse-caregiver	Non-spouse caregiver	p	d
Physical health	2.42 (0.74)	2.42 (0.73)	0.98	0	2.40 (0.74)	2.71 (0.73)	0.04*	-0.42
Energy	2.51 (0.74)	2.69 (0.74)	0.22	-0.24	2.89 (0.84)	2.92 (0.84)	0.83	-0.03
Mood	2.65 (0.70)	2.75 (0.69)	0.43	-0.14	2.65 (0.78)	2.85 (0.77)	0.16	-0.25
Living situation	3.14 (0.50)	3.04 (0.50)	0.52	0.20	2.95 (0.77)	3.08 (0.76)	0.45	-0.16
Memory	3.14 (0.50)	2.16 (0.68)	0.97	1.66	2.85 (0.68)	2.69 (0.67)	0.41	0.23
Family	3.22 (0.47)	3.14 (0.47)	0.43	0.17	3.14 (0.70)	3.02 (0.70)	0.37	0.17
Marriage	3.26 (0.57)	1.04 (0.57)	< 0.001^†^	3.89	3.10 (0.72)	2.16 (0.71)	0.01*	1.31
Friends	2.83 (0.69)	2.77 (0.69)	0.73	0.08	2.93 (0.74)	3.24 (0.74)	0.04*	-0.41
Self as a whole	2.53 (0.65)	2.85 (0.65)	0.02*	-0.39	2.73 (0.70)	2.87 (0.70)	0.21	-0.20
Ability to do chores	2.47 (0.82)	2.67 (0.82)	0.22	-0.24	3.26 (0.70)	3.20 (0.70)	0.72	0.08
Ability to do things for fun	2.34 (0.86)	2.59 (0.85)	0.13	-0.29	2.75 (0.85)	2.69 (0.85)	0.81	0.07
Money	2.14 (0.79)	2.36 (0.79)	0.20	-0.27	2.32 (0.80)	2.47 (0.80)	0.50	-0.18
Life as a whole	2.69 (0.66)	2.96 (0.65)	0.03*	-0.41	2.63 (0.73)	2.87 (0.72)	0.08	-0.33
Total score	34.4 (4.7)	33.6 (4.8)	0.29	0.16	36.4 (5.1)	36.7 (6.6)	0.65	-0.05

Data presented as mean (standard deviation).

d = Cohen’s d; PwAD = people with Alzheimer’s disease; QoL = quality of life.

* p < 0.05; ^†^ p < 0.001.

**Table 3:** Differences between self-report QoL domain ratings for spouse and non-spouse PwAD and caregivers

QoL	Spouse-PwAD	Spouse-caregiver	p	d	Non-spouse-PwAD	Non-spouse-caregiver	p	d
Physical health	2.43 (0.73)	2.41 (0.73)	0.89	0.02	2.43 (0.67)	2.71 (0.89)	0.08	-0.35
Energy	2.51 (0.73)	2.90 (0.84)	0.01*	-0.49	2.69 (0.71)	2.92 (0.90)	0.21	-0.28
Mood	2.65 (0.69)	2.65 (0.77)	1	0	2.76 (0.63)	2.86 (0.73)	0.49	-0.14
Living situation	3.14 (0.50)	2.96 (0.76)	0.11	0.28	3.04 (0.61)	3.08 (0.73)	0.76	0.05
Memory	2.14 (0.67)	2.86 (0.67)	< 0.000^†^	-1.07	2.16 (0.87)	2.69 (0.79)	0.004^†^	-0.63
Family	3.22 (0.46)	3.14 (0.70)	0.43	0.13	3.14 (0.50)	3.02 (0.72)	0.37	0.19
Marriage	3.27 (0.56)	3.10 (0.71)	0.17	0.26	1.04 (1.45)	2.16 (1.65)	0.001^†^	-0.72
Friends	2.84 (0.68)	2.94 (0.74)	0.39	-0.14	2.78 (0.77)	3.24 (0.63)	0.001^†^	-0.65
Self as a whole	2.53 (0.64)	2.73 (0.70)	0.08	-0.29	2.86 (0.70)	2.88 (0.72)	0.89	-0.02
Ability to do chores	2.47 (0.81)	3.27 (0.70)	< 0.000^†^	-1.05	2.67 (0.65)	3.2 (0.73)	< 0.000^†^	-0.76
Ability to do things for fun	2.35 (0.85)	2.76 (0.85)	0.01*	-0.48	2.59 (0.91)	2.69 (0.96)	0.60	-0.10
Money	2.14 (0.79)	2.33 (0.80)	0.22	-0.23	2.37 (0.72)	2.47 (0.73)	0.49	-0.13
Life as a whole	2.69 (0.65)	2.63 (0.72)	0.69	0.08	2.96 (0.57)	2.88 (0.75)	0.54	0.12
Total score	34.4 (4.7)	36.4 (5.1)	0.01*	-0.40	33.6 (4.8)	36.7 (6.6)	0.006^†^	-0.54

Data presented as mean (standard deviation).

d = Cohen’s d; PwAD = people with Alzheimer’s disease; QoL = quality of life.

* p < 0.05; ^†^ p < 0.01.

## Discussion

This study investigated the QoL of PwAD and caregivers, analyzing the influence of different types of kinship on self-report ratings. Commonly, studies that investigate QoL between PwAD and their caregivers include several kinds of caregivers such as spouse, adult children, and other relatives, and do not take into consideration the differences across types of kinship, treating caregivers as a single group.^1,2^ We therefore decided to compare two groups of kinship (spouse vs. non-spouse) in PwAD/caregiver dyads to better understand their influence on QoL ratings and domains.

### Differences between caregivers’ self-report QoL domains according to degree of kinship

Comparison of the clinical and sociodemographic characteristics of the two caregiver groups showed that spouse-caregivers were older and had higher burden levels. Fauth et al.^18^ reported that emotional closeness has an impact on caregiver well-being and, consequently, on the type of care provided. Overall, spouse-caregivers, especially female spouse-caregivers, may consider the tasks of caring for their spouse to be part of their marital commitment, and commonly assume care without help. Consequently, taking on different roles such as caregiver, wife, mother, and lover, may cause conflicts among these disparate roles. Therefore, the caregiving routine may morph the marital intimacy into a parent-child relationship. Bearing in mind that the relationship between spouses is closer, spouse-caregivers may be more overwhelmed by the change in their roles, and so, the change in the relationship may impose a greater burden of care.^7^

We expected that spouse-caregivers would have lower QoL than non-spouse caregivers. The non-spouse caregivers did have a slightly higher QoL, although the difference in the QoL total score was not practical or statistically significant. Nonetheless, when QoL was analyzed by domains, non-spouse caregivers seemed to have more friends and perceived their own physical health as better than spouse-caregivers. Non-spouse caregivers commonly receive help from other family members, and provide less care than the spouse-caregiver, who are often the primary caregiver.^3^ In addition, it is possible that the fact that spouse-caregivers were older may have influenced the way they perceived their physical health, since they are often frail themselves.^3^ Studies have shown that better caregiver mental and physical health is consistently associated with better QoL.^4^ This finding underlines the potential value of studying the differences between different caregiver groups in order to attend to their needs effectively.

### Differences between self-report QoL ratings of PwAD and caregivers

Analysis of the total QoL scores showed that caregivers’ self-report QoL was higher than PwAD’s self-report QoL in both groups. Several studies have reported higher QoL scores for PwAD than for caregivers, because of the presence of impaired awareness of disease.^6,19,20^ Conversely, we found that both groups of caregivers had better QoL than both groups of PwAD. The most likely explanation may lie in the fact that our sample was recruited at a specialized outpatient clinic that offers many services for caregivers, such as psychoeducational groups and physician appointments, if necessary. According to Santos et al.,^20^ caregivers’ subjective experiences of the different domains of QoL are more predictive of caregivers’ self-report QoL than PwAD-related variables. Furthermore, we may suppose that the clinical symptoms of AD were possibly not perceived as uncontrollable by the caregivers assessed in this study. Our sample of PwAD mostly had mild disease severity and lower levels of depression and caregivers’ QoL may be influenced by the context at each stage of the disease.^6^

Furthermore, it has been suggested that social and interpersonal factors may also play a stronger role in evaluations of QoL.^21^ Moreover, interpretations related to QoL may be ambiguous when PwAD and caregivers are asked to answer the same questions. In our sample, we found that spouse dyads differed in fewer QoL domains than non-spouse dyads. Discrepancies between the members of spouse dyads were only found in two domains (energy and ability to do things for fun), whereas non-spouse dyads had discrepancies in marriage, friends, and ability to do chores. Also, both groups had discrepancies for memory, as expected. We may thus perceive that spouses become closer and develop mechanisms to cope with difficulties together, thereby exhibiting more agreement about QoL when compared to other kinds of caregivers. So, a close relationship and strong feelings in the relationship facilitate a more positive evaluation of a situation and help to adjust expectations for both members of the dyad.^21^

### Limitations

Our study has some limitations. First, QoL was evaluated in a convenience sample of patients with mild to moderate levels of Alzheimer’s disease; no analyses were conducted to assess disease severity differences between the two groups. In future studies, comparison of QoL by kinship of caregivers should be investigated in studies controlled for severity of dementia, because judgments about what is important to evaluate QoL may change over the course of dementia progression.^1^ Second, although levels of burden were assessed, no analysis was conducted to investigate the influence of burden on caregivers’ QoL. In our study, the majority of participants had mild disease and, maybe because of this, caregivers’ burden was low. Commonly, caregivers with moderate levels of burden experience difficulties that negatively affect their QoL.^11^ In future studies, we should include PwAD with more severe disease levels to further investigate the relationship between burden and QoL. It would also be important to explore a range of different levels of support and help received by caregivers from other family members. Having help may influence how caregivers perceive the role of caring, and may influence their own QoL. Third, evaluation of depressive and anxious symptoms among caregivers should be investigated in future studies, because these symptoms can impact QoL. Also, further studies should investigate the influence of psychoeducational interventions on QoL.

### Clinical implications and future directions

Quality of life is a multidimensional concept associated with expectations, concerns, and goals, including social, physical, and psychological functioning. Because QoL is a complex concept, each group may evaluate it differently. The findings of this study help to decide whether promoting specific services can be recommended for each kind of kinship. Also, the study findings would allow providers and clinicians to promote good QoL both in PwAD and their caregivers, taking into consideration the characteristics and demands of each group.

## Conclusion

The investigation of spouse and non-spouse caregivers’ QoL did not detect a significant difference in QoL as a whole. On the other hand, when QoL was analyzed by domains, there were differences between spouse and non-spouse caregiver groups. Our findings show that different groups of caregivers have specific appraisals and perceptions regarding what is important to evaluate with regard to their own QoL. Even though the two groups did not differ significantly when QoL was evaluated as a single construct, each group considered different specific QoL-related domains to be important. Quality of life is complex and should be evaluated in domains and not globally.
